# Physical training for asthma

**DOI:** 10.1590/1516-3180.20141323T1

**Published:** 2014-04-14

**Authors:** Kristin V. Carson, Madhu G. Chandratilleke, Joanna Picot, Malcom P. Brinn, Adrian J. Esterman, Brian J. Smith

## Abstract

**BACKGROUND:**

People with asthma may show less tolerance to exercise due to worsening asthma symptoms during exercise or other reasons such as deconditioning as a consequence of inactivity. Some may restrict activities as per medical advice or family influence and this might result in reduced physical fitness. Physical training programs aim to improve physical fitness, neuromuscular coordination and self confidence. Subjectively, many people with asthma report that they are symptomatically better when fit, but results from trials have varied and have been difficult to compare because of different designs and training protocols. Also, as exercise can induce asthma, the safety of exercise programmes needs to be considered.

**OBJECTIVE:**

To gain a better understanding of the effect of physical training on the respiratory and general health of people with asthma, from randomised trials.

**METHODS:**

*Search methods:* We searched the Cochrane Airways Group Specialised Register of trials up to January 2013.

*Selection criteria:* We included randomised trials of people over eight years of age with asthma who were randomised to undertake physical training or not. Physical training had to be undertaken for at least 20 minutes, two times a week, over a minimum period of four weeks.

*Data collection and analysis:* Two review authors independently assessed eligibility for inclusion and undertook risk of bias assessment for the included studies.

**MAIN RESULTS:**

Twenty-one studies (772 participants) were included in this review with two additional 2012 studies identified as ‘awaiting classification’. Physical training was well tolerated with no adverse effects reported. None of the studies mentioned worsening of asthma symptoms following physical training. Physical training showed marked improvement in cardiopulmonary fitness as measured by a statistically and clinically significant increase in maximum oxygen uptake (mean difference (MD) 4.92 mL/kg/min; 95% confidence interval (CI) 3.98 to 5.87; P < 0.00001; 8 studies on 267 participants); however, no statistically significant effects were observed for forced expiratory volume in 1 second (FEV1), forced vital capacity (FVC), minute ventilation at maximal exercise (VEmax) or peak expiratory flow rate (PEFR). Meta-analysis of four studies detected a statistically significant increase in maximum heart rate, and following a sensitivity analysis and removal of two studies significance was maintained (MD 3.67 bpm; 95% CI 0.90 to 3.44; P = 0.01). Although there were insufficient data to pool results due to diverse reporting tools, there was some evidence to suggest that physical training may have positive effects on health-related quality of life, with four of five studies producing a statistically and clinically significant benefit.

**AUTHORS’ CONCLUSIONS:**

This review demonstrated that physical training showed significant improvement in maximum oxygen uptake, though no effects were observed in other measures of pulmonary function. Physical training was well tolerated among people with asthma in the included studies and, as such, people with stable asthma should be encouraged to participate in regular exercise training, without fear of symptom exacerbation. More research is needed to understand the mechanisms by which physical activity impacts asthma management.

**This is the abstract of a Cochrane Review published in the Cochrane Database of Systematic Reviews (CDSR) 2013, issue 9, Art. No. CD001116. DOI:** 10.1002/14651858.CD001116.pub4 (http://onlinelibrary.wiley.com/doi/10.1002/14651858.CD001116.pub4/abstract).

**The full text is available from:** http://onlinelibrary.wiley.com/doi/10.1002/14651858.CD001116.pub4/abstract;jsessionid=EA0BC34385812E99C5D313683EE0D160.f03t04.

**The abstract is also available in the Portuguese, French and Spanish languages from:** http://summaries.cochrane.org/CD001116/physical-training-for-asthma.
